# Integrin subunit beta 6 is a potential diagnostic marker for acute kidney injury in patients with diabetic kidney disease: a single cell sequencing data analysis

**DOI:** 10.1080/0886022X.2024.2409348

**Published:** 2024-10-02

**Authors:** Congcong Yao, Ziwei Li, Hongshuang Su, Keke Sun, Qihui Liu, Yan Zhang, Lishuang Zhu, Feng Jiang, Yaguang Fan, Songtao Shou, Heng Wu, Heng Jin

**Affiliations:** a Department of Emergency Medicine, Tianjin Medical University General Hospital, Tianjin, China; b Department of Critical Care Medicine, Tianjin Medical University General Hospital, Tianjin, China; c Department of Ophthalmology, Tianjin Medical University General Hospital, Tianjin, China; d Tianjin Key Laboratory of Lung Cancer Metastasis and Tumor Microenvironment, Tianjin Lung Cancer Institute, Tianjin Medical University General Hospital, Tianjin, China

**Keywords:** Diabetic kidney disease, acute kidney injury, single-cell RNA sequencing, renal tubular epithelial cells, diagnostic markers, therapeutic target

## Abstract

**Background:**

Diabetic kidney disease (DKD), a prevalent complication of diabetes mellitus, is often associated with acute kidney injury (AKI). Thus, the development of preventive and therapeutic strategies is crucial for delaying the progression of AKI and DKD.

**Methods:**

The GSE183276 dataset, comprising the data of 20 healthy controls and 12 patients with AKI, was downloaded from the Gene Expression Omnibus (GEO) database to analyze the AKI group. For analyzing the DKD group, the GSE131822 dataset, comprising the data of 3 healthy controls and 3 patients with DKD, was downloaded from the GEO database. The common differentially expressed genes (DEGs) in renal tubular epithelial cells (TECs) were subjected to enrichment analyses. Next, a protein-protein interaction (PPI) network was constructed using the Search Tool for the Retrieval of Interacting Genes database to analyze gene-related regulatory networks. Finally, the AKI animal models and the DKD and AKI cell models were established, and the reliability of the identified genes was validated using quantitative real-time polymerase chain reaction analysis.

**Results:**

Functional analysis was performed with 40 common DEGs in TECs. Eight hub genes were identified using the PPI and gene-related networks. Finally, validation experiments with the *in vivo* animal model and the *in vitro* cellular model revealed the four common DEGs. Four DEGs that share molecular mechanisms in the pathogenesis of DKD and AKI were identified. In particular, the expression of Integrin Subunit Beta 6(*ITGB6)*, a hub and commonly upregulated gene, was upregulated in the *in vitro* models.

**Conclusion:**

*ITGB6* may serve as a biomarker for early AKI diagnosis in patients with DKD and as a target for early intervention therapies.

## 1. Introduction

Diabetic kidney disease (DKD), a long-term complication of diabetes, leads to microvascular complications in the kidneys that occur in both type I diabetes mellitus and type II diabetes mellitus (T2DM) [1]. The global number of diabetes cases was as high as 537 million in 2021 and is predicted to increase to 580 and 700 million by 2030 and 2045, respectively [2–4]. More than 50% of patients with end-stage renal disease (ESRD) have diabetes [5]. The number of DKD cases in China has increased from 17.34 million in 1990 to 31.65 million in 2019 [6]. Additionally, a meta-analysis indicated that the prevalence of DKD among patients with T2DM in China is 21.8% [7].

Previous studies have demonstrated that diabetes is an independent risk factor for acute kidney injury (AKI) [8–10]. AKI is a clinical syndrome characterized by a rapid decline in kidney function. In China, the annual incidence of AKI is approximately 13.3 million cases with 1.7 million deaths attributed to AKI [11,12]. The incidence of AKI among hospitalized patients in high-income countries is up to 20%, affecting approximately 50% and 25% of adult and pediatric patients in intensive care, respectively [13,14]. Various factors, such as surgeries, medications, and sepsis/septic shock increase the incidence of AKI in patients with diabetes [15–17]. AKI is a risk factor for the occurrence and progression of chronic kidney disease (CKD) and ESRD [18]. The risk of developing or progressing to CKD, ESRD, cardiovascular events, and mortality is 2.67-fold, 4.81-fold, 38%, and 1.80-fold higher in patients with AKI [19]. Approximately 30%–70% of patients with AKI develop CKD within 1 year with approximately 17% of cases progressing to ESRD [20]. Additionally, the 5-year rehospitalization rate is 32.4% with more than 2 million AKI-related deaths annually worldwide [21]. Patients are primarily managed with dialysis and supportive care due to the lack of early prevention and effective treatments as the mechanisms underlying AKI occurrence and progression have not been elucidated [22,23]. Thus, AKI is a major global public health issue, contributing to increased medical and financial burdens for patient families and society [24].

DKD and AKI are major contributors to ESRD development and involve intricate pathological and physiological processes. The increased prevalence of DKD and AKI with the potential for DKD progressing to AKI warrants the elucidation of the mechanisms underlying the correlation between DKD and AKI. The elucidation of the shared mechanisms between DKD and AKI is crucial for early diagnosis and treatment. Advancements in RNA sequencing technology and bioinformatics analysis methods have markedly improved the sensitivity, accuracy, and efficiency of single-cell RNA sequencing (scRNA-seq) [25]. Compared with conventional RNA sequencing, scRNA-seq has the advantages of analyzing gene expression in individual cells and comprehensively revealing molecular targets and signaling pathways of human diseases at the single-cell level [26–30]. This novel technology has been applied to study various kidney diseases [31–34].

**Figure 1.**
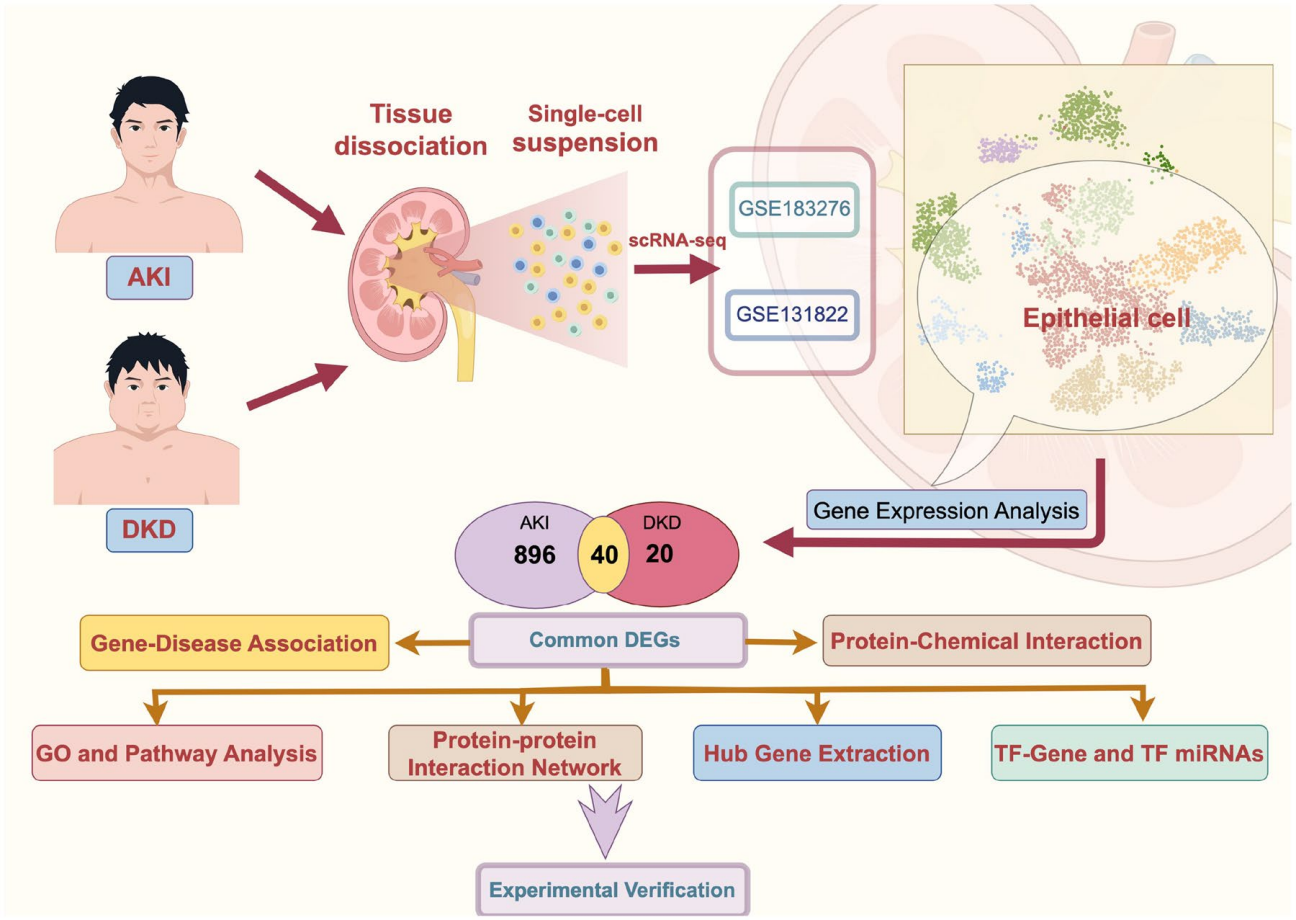
Schematic illustration of the overall general workflow of this study.

In this study, the scRNA-seq data were used to comparatively analyze the transcriptomic features of renal tubular epithelial cells (TECs) between AKI and DKD. The identification of common differentially expressed genes (DEGs) and pathways between AKI and DKD enables the elucidation of the mechanisms shared between AKI and DKD, as well as identifying potential therapeutic targets. Thus, these findings have research value and clinical significance for the prevention and treatment of DKD that can progress to AKI. The flowchart of the study procedure is shown in Figure 1.

## 2. Materials and methods

### 2.1. Study design and data collection

All eligible datasets were obtained from the Gene Expression Omnibus (GEO) database (https://www.ncbi.nlm.nih.gov/geo/). The relevant gene expression datasets were searched using keywords related to AKI and DKD. The selection criteria were as follows: (1) scRNA-seq datasets; (2) data of renal tissue specimens; and (3) inclusion of data from adult individuals. Based on these criteria, the following two datasets were included in this study: GSE183276 [35] and GSE131822 [32]. GSE183276, which is annotated on platform GPL24676, comprised the renal scRNA-seq data of 20 healthy controls and 12 patients with AKI. Meanwhile, GSE131822, which is also annotated on platform GPL24676, comprised the renal scRNA-seq data of 3 healthy controls and 3 patients with DKD.

### 2.2. Single-cell analysis and identification of DEGs

In this study, single-cell transcriptome analysis was conducted on data files downloaded from the GEO public database using the Seurat package (version 4.3.0) in R software (version 4.2.2) [36]. Initially, exon results from each sample were processed as independent samples, with the expression profiles read using parameters set to a minimum of 3 cells and 500 features. High-quality single-cell datasets were curated through quality control analysis with the FindVariableFeatures function, applying criteria of nFeature_RNA > 500 and percentage.mt < 5. Subsequently, data normalization was performed using the NormalizeData function, followed by the selection of the top 2000 most variable genes across cells. Ten genes with the highest normalized variance were identified, providing key features for subsequent analysis. Principal component analysis (PCA) was then conducted on these highly variable genes using the ScaleData and RunPCA functions, with the number of principal components (npcs) set to 20. The optimal number of PCs was determined using ElbowPlot and JackStraw methods, with 15 PCs being selected for further analysis. Based on the selected PC values, clustering (Cluster), UMAP, and tSNE values were obtained using the RunUMAP, RunTSNE, FindNeighbors, and FindClusters functions, respectively. A resolution parameter of 0.8 was set for cluster analysis. Cell subtypes within the clusters were annotated according to relevant literature, and the number of cell samples in each subtype was quantified [32, 35, 37]. Furthermore, DEGs specific to each cell subtype were identified using the FindMarkers method, laying the foundation for further biological analysis(reference link: https://satijalab.org/seurat/articles/integration_introduction.html).

### 2.3. Functional annotation and pathway enrichment analysis

To further elucidate the functions of common DEGs, the ‘cluster profiler’ package (version 4.6.2) in R software was used for Gene Ontology (GO) annotation and Kyoto Encyclopedia of Genes and Genomes (KEGG) pathway enrichment analysis [38]. The GO terms were biological processes (BP), cellular components (CC), and molecular functions (MF). Functional enrichment analysis of important modules was performed for both GO and KEGG pathways. The enrichment was considered significant at *p* < 0.05.

### 2.4. Protein-protein interaction (PPI) network construction

Based on the common DEGs, a PPI network was constructed using the Search Tool for the Retrieval of Interacting Genes (STRING) database (https://cn.string-db.org/) and visualized using Cytoscape 3.10.1 [39]. The confidence score was set to the median value (confidence score = 0.4). CytoHubba, a plugin for Cytoscape software to identify hub genes, measures nodes based on their network characteristics, enabling the identification of important nodes in biological networks [40]. Additionally, the CytoHubba plugin was used to identify hub genes using seven common algorithms Mathews correlation coefficient (MCC), maximum neighborhood component (MNC), degree, closeness, radiality, edge percolated component (EPC), and density of MNC (DMNC) algorithms) for evaluation and selection.

### 2.5. Gene-related regulatory network analysis

The NetworkAnalyst online tool was used to construct the gene regulatory network of hub gene-transcription factor (hub gene-TF) interaction [41]. To construct the hub gene-TF network, the TF and gene target data were obtained from the ENCODE chromatin immunoprecipitation-sequencing data [42]. The data collected from the RegNetwork database were used to construct the TF-miRNA co-regulatory interaction network. Additionally, data information for the gene-disease regulatory network was sourced from the DisGeNET database, which is a comprehensive database of gene-disease associations, synthesizing relationships from various biomedical aspects of illnesses. These data highlight the emerging insights into human genetic disorders [43]. Further, the gene-disease relationship was examined using NetworkAnalyst to uncover diseases with common DEGs and their chronic complications. NetworkAnalyst was used to construct the protein-chemical interaction network. Protein-chemical interaction data were sourced from the Comparative Toxicogenomics Database, which contains interactions between chemicals and genes documented in the literature [44].

### 2.6. Establishment of animal models

The models of cecal ligation and puncture (CLP)-induced septic AKI and rhabdomyolysis-induced AKI (RM-AKI; established using glycerol) were established. C57BL/6 J mice (aged 6–8 weeks) were obtained from the Experimental Animal Center of Tianjin Medical University. Mice were housed in separate cages under the following conditions: temperature, 20–26 °C; access to food and water, *ad libitum*; circadian cycle, 12-h light/dark cycle. After adaptive feeding for 6 days, mice were randomly assigned into the following three groups (3 mice/group): control group, CLP group, and glycerol group. Mice in the CLP group were subjected to CLP and sacrificed at 24 h post-CLP surgery. To establish the RM-AKI model, the hindlimb medial and lateral femoral muscles of mice in the glycerol group were injected with 50% glycerol in physiological saline (10 mL/kg bodyweight), and the mice were sacrificed at 48 h post-glycerol injection. The kidney tissues were collected for subsequent experiments.

### 2.7. Cell culture

The human TEC line (HK-2 cells) was purchased from the cell bank of Shanghai Enzyme Research Biotechnology Co., Ltd. HK-2 cells were maintained by the Emergency Medicine Laboratory of Tianjin Medical University General Hospital and further resuscitated and passaged in the Emergency Medicine Laboratory of Tianjin Medical University General Hospital. HK-2 cells were cultured in Dulbecco’s modified Eagle medium/F12 medium supplemented with 10% fetal bovine serum at 37 °C in a 5% CO_2_ humidified incubator. The cells were seeded in 6-well plates (1 × 10^6^ cells/well), stimulated with lipopolysaccharide (LPS) (100 ng/mL, 1 μg/mL, or 10 μg/mL) for 24 h, and incubated with a high glucose concentration (30 mmol/L) for 24 and 48 h [45–47]. Cells were then harvested and subjected to total RNA extraction and quantitative real-time polymerase chain reaction (qRT-PCR) analysis (primer sequences are shown in Supplementary Table 1).

### 2.8. RNA extraction and qRT-PCR analysis

Total RNA was extracted from cells using the SPARKeasy Improved Tissue/Cell RNA Kit, following the manufacturer’s instructions. RNA (1 µg) was reverse-transcribed into complementary DNA using the FastKing gDNA Dispelling RT SuperMix kit for qRT-PCR analysis. GAPDH and β-actin were used as an internal reference. The relative expression levels of the target genes were calculated using the 2^−ΔΔCt^ method.

### 2.9. Statistical analysis

All statistical analyses and graphing were performed using R software (version 4.1.2) or GraphPad Prism 9.5.1. The normal distribution of data was confirmed using the Shapiro-Wilk test. The normally distributed variables were expressed as mean ± standard deviation. Meanwhile, the non-normally distributed continuous variables were expressed as median and interquartile range. Additionally, the means between the groups were compared using one-way analysis of variance. Differences were considered significant at *p* < 0.05 (*p < 0.05, **p < 0.01, ***p < 0.001 for all numerical values).

## 3. Results

### 3.1. Fifteen cell types were identified in the AKI cohort

**Figure 2.**
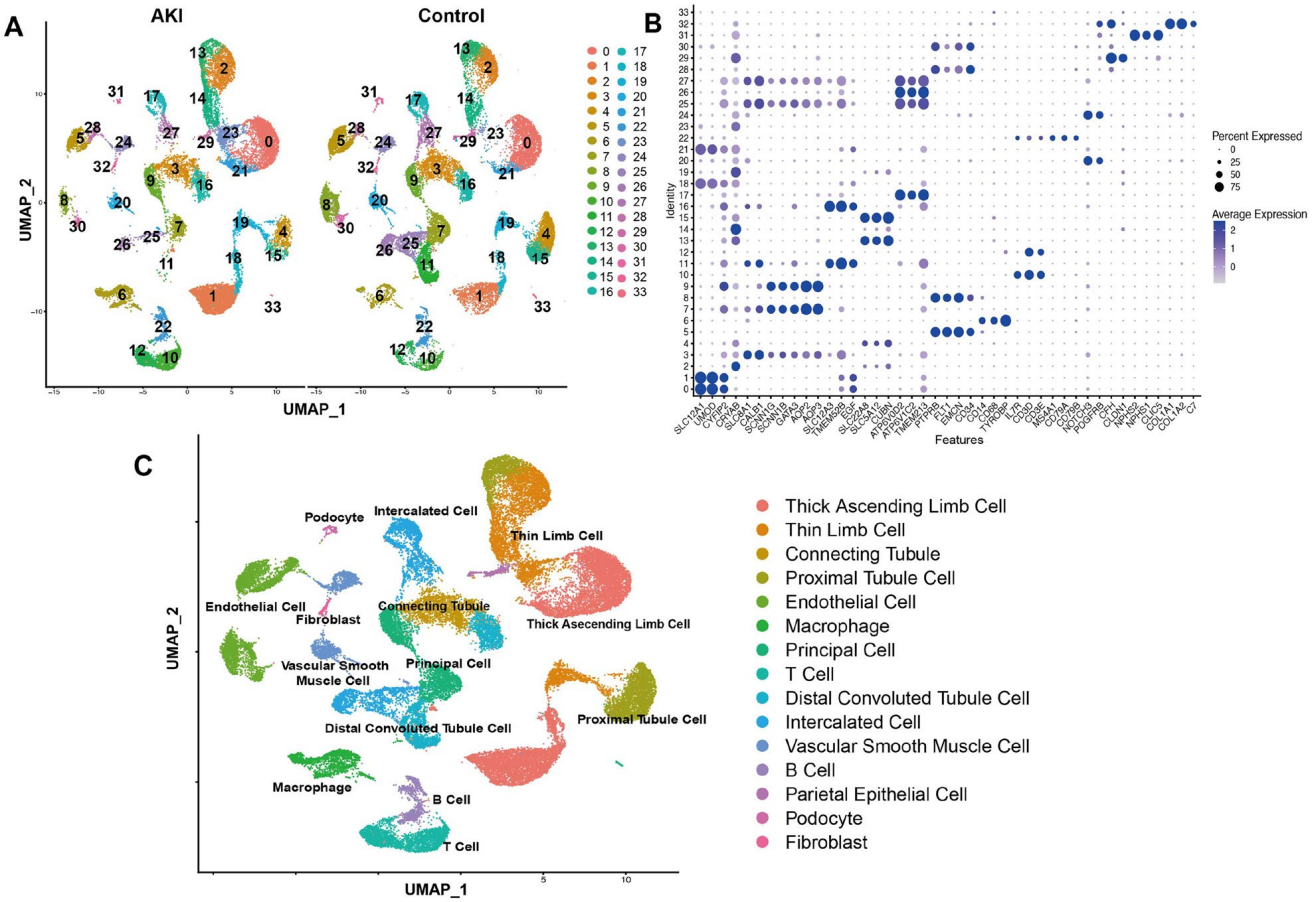
Single-cell RNA sequencing (scRNA-seq) data analysis of renal cell lineage in patients with AKI and healthy individuals. (A) The uniform manifold approximation and projection (UMAP) plots were used to analyze the renal cell lineage of patients with AKI and healthy subjects, resulting in the identification of 33 subgroups within the kidney. Each color within the plots represents a distinct subgroup. (B) Bubble plots were used to illustrate selected marker genes for each subgroup. The size of each bubble corresponds to the percentage of each marker gene within the respective cell type, while the color intensity signifies the average expression level of each marker gene within the cell type. (C) The UMAP plots were used to identify 15 distinct cell clusters in the kidney with each cell represented by a specific color denoting a different subgroup. AKI represents patients with AKI; control represents healthy subjects.

After the screening of the GSE183276 dataset, the data of individuals with a history of diabetes and those aged over 40 were excluded. The dataset comprised the data of 20 healthy controls and 12 patients with AKI. After screening, the data of 20 healthy controls and 7 patients with AKI (encompassing the data of 21,650 and 20,717 cells in the healthy control and AKI groups, respectively) were included in the analysis. Stringent quality control measures were implemented, and thorough data processing was conducted to develop a single-cell transcriptional atlas. These cells were effectively categorized into 33 major clusters (Figure 2A). To provide further insights, the expression of marker genes in each cell type was visualized (Figure 2B, Supplementary Table 2). After cross-referencing the identified marker genes with established cell type-specific markers, the cell clusters were categorized into the following 16 distinct types: proximal tubule cells, distal convoluted tubule cells, thin limb cells, thick ascending limb cells, principal cells, intercalated cells, collecting ducts, endothelial cells, B cells, T cells, podocytes, macrophages, fibroblasts, vascular smooth muscle cells, and parietal epithelial cells (Figure 2C).

### 3.2. Twelve cell types were identified in the DKD cohort

**Figure 3.**
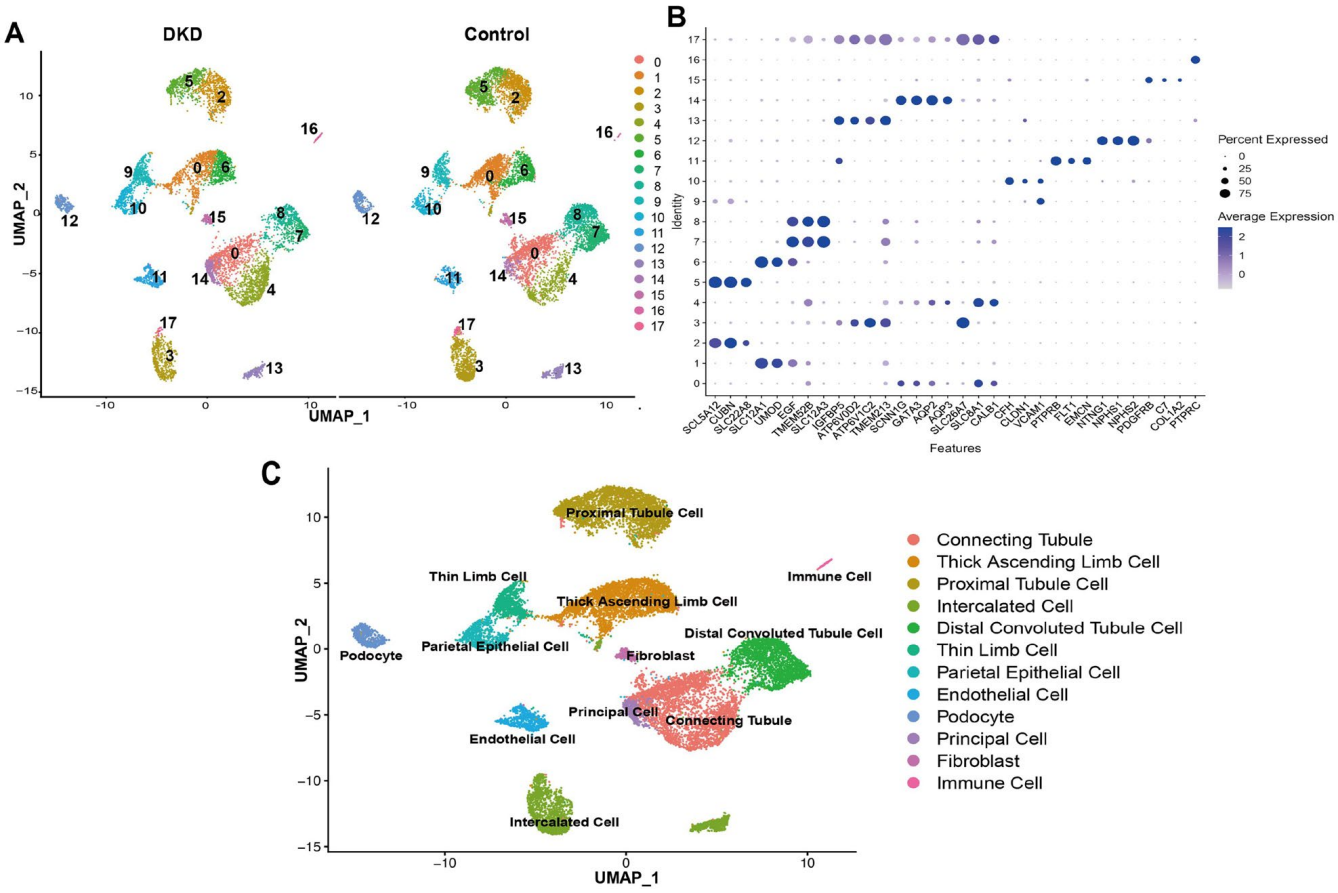
Single-cell RNA sequencing (scRNA-seq) data analysis of renal cell lineage in patients with DKD and healthy individuals. (A) The uniform manifold approximation and projection (UMAP) plots were used to identify the renal cell lineage of patients with DKD and healthy subjects, resulting in the identification of 18 subgroups within the kidney. Each color within the plots represents a distinct subgroup. (B) Bubble plots were used to illustrate selected marker genes for each subgroup. The size of each bubble corresponds to the percentage of each marker gene within the respective cell type, while the color intensity signifies the average expression level of each marker gene within the cell type. (C) The UMAP plots were used to distinguish 12 different cell clusters in the kidney with each cell represented by a specific color denoting a different subgroup. DKD indicates patients with DKD; control represents healthy subjects.

The GSE131822 dataset, comprising the data of 3 healthy controls and 3 patients with DKD, was used to obtain single-cell objects for analysis. In this study, 11,621 cells were identified with 32,716 features in the healthy control group, while 7,723 cells were identified with 29,004 features in the DKD group. The clustered analysis revealed 18 major cell populations (Figure 3A). Analysis of the expression profiles of marker genes revealed distinct cellular identities (Figure 3B, Supplementary Table 2). Comparative analysis of identified marker genes and established cell type-specific markers enabled the categorization of the cell groups within the clusters into the following 12 distinct cell types: proximal tubule cells, distal convoluted tubule cells, thin limb cells, thick ascending limb cells, principal cells, intercalated cells, collecting duct cells, endothelial cells, immune cells, podocytes, fibroblast cells, and parietal epithelial cells (Figure 3C). This comprehensive categorization enhances our understanding of cellular heterogeneity and provides valuable insights into the pathophysiology of DKD.

### 3.3. Role of TECs in AKI and DKD

**Figure 4.**
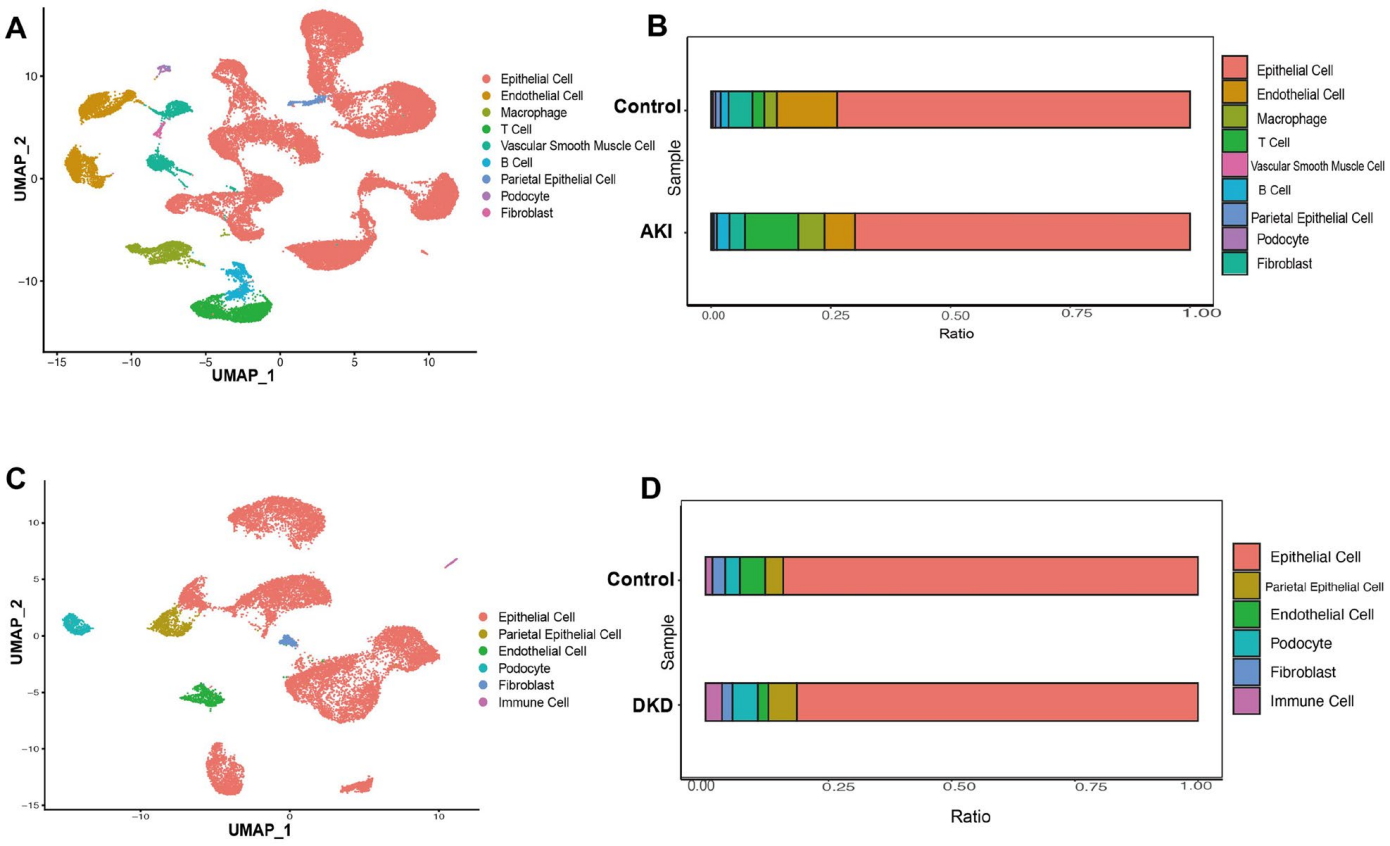
The proportion of renal TECs in patients with AKI and those with DKD. (A) The UMAP plots revealed nine distinct cell clusters in the kidney of the AKI group. Each cell is represented by a color indicating different subgroups. (B) Bar graphs show the percentage of cell clusters in the kidneys of the AKI and control groups. (C) The UMAP plots revealed six distinct cell clusters in the kidney of the DKD group. Each cell is represented by a color indicating different subgroups. (D) Bar graphs show the percentage of cell clusters in the kidneys of the DKD and control groups. Each block represents a different subject, and the length of the block is proportional to the number of cells. Different cell clusters are indicated using different colors.

Through extensive literature review and experimental studies, we have determined that renal TECs, which constitute a major component of the renal cortex, are the primary target cells in the onset and progression of AKI. TECs are reported to be highly susceptible to injury in both AKI and DKD. Renal tubular cell death is the hallmark of AKI [48]. Additionally, tubular damage is reported to occur early in DKD and may play a key role in the progression of kidney disease [49]. Subsequent analysis led to the refinement of cell populations within the AKI cohort, revealing the following nine distinct cell types: epithelial cells(proximal tubule cells, distal convoluted tubule cells, thin limb cells, thick ascending limb cells, principal cells, intercalated cells, collecting duct cells), endothelial cells, B cells, T cells, podocytes, macrophages, fibroblast cells, vascular smooth muscle cells, and parietal epithelial cells (Figure 4A).

Comparative analysis demonstrated that the refined cell population markedly varied between the AKI and control groups. In particular, the percentage of TECs, endothelial cells, podocytes, vascular smooth muscle cells, and parietal epithelial cells in the AKI group was lower than that in the healthy control group (Figure 4B). Meanwhile, the percentage of T cells, B cells, and macrophages was downregulated in the AKI group. The proportion of TECs was significantly downregulated in the AKI group, suggesting that these cells are involved in the pathogenesis of AKI. These findings indicate the dynamic changes at the cellular level in AKI.

In the DKD group, the following six distinct cell types were identified: epithelial cells (proximal tubule cells, distal convoluted tubule cells, thin limb cells, thick ascending limb cells, principal cells, intercalated cells, collecting duct cells), endothelial cells, immune cells, podocytes, fibroblast cells, and parietal epithelial cells (Figure 4C). The proportion of TECs and endothelial cells in the DKD group was significantly lower than that in the healthy control group, indicating the critical role of TECs in the pathogenesis of DKD (Figure 4D).

### 3.4. Common DEGs in TECs between AKI and DKD

**Figure 5.**
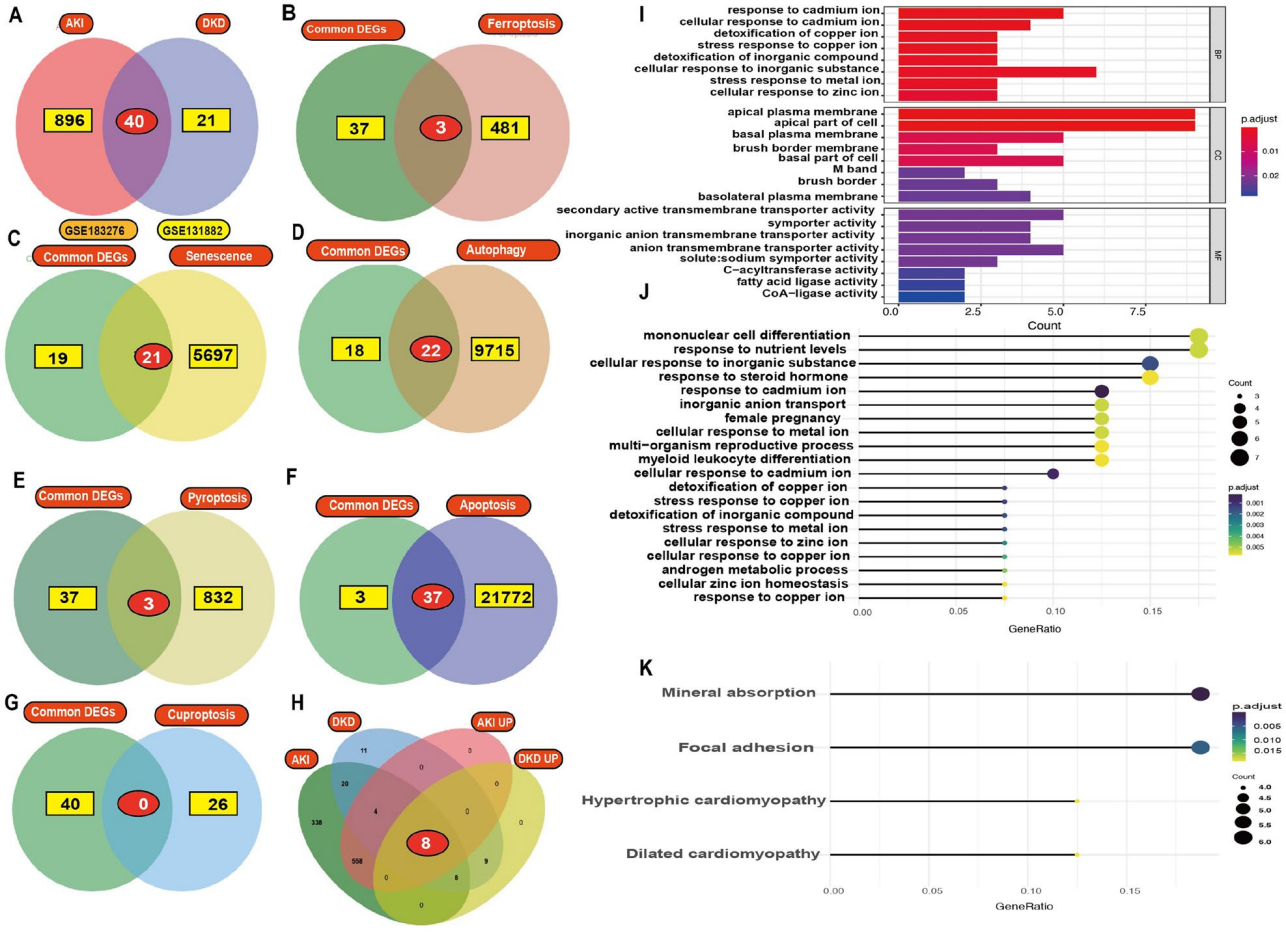
The Venn diagram and functional enrichment analysis of the common DEGs in renal TECs. (A) This study used two datasets AKI (GSE183276) and DKD(GSE131822) datasets and identified 40 common DEGs. (B) From the FerrDb database, 484 genes associated with ferroptosis were identified. (C) From the GeneCards database, 5718 genes associated with senescence were identified. (D) From the GeneCards database, 9737 genes associated with autophagy were identified. (E) From the GeneCards database, 835 genes associated with pyroptosis were identified. (F) From the GeneCards database, 21809 genes associated with apoptosis were identified. (G) From the GeneCards database, 26 genes associated with cuproptosis were identified. (H) In total, 8 common upregulated DEGs and 20 common downregulated DEGs were identified between the AKI and DKD datasets. (I) Functional enrichment of common DEGs in the gene Ontology (GO) terms biological process, cellular component, and molecular function. (J) Functional enrichment of common DEGs in the GO term biological processes. (K) Kyoto Encyclopedia of Genes and Genomes pathway enrichment of common DEGs.

The percentage of TECs in the AKI and DKD groups was significantly lower than that in the healthy control group. To elucidate the molecular differences between AKI and DKD, the transcriptional changes in TECs were analyzed. The R software package ‘Seurat’ was used to analyze DEGs, resulting in the identification of 936 DEGs (|log2fold-change|> 0.25 and *p* < 0.05) in TECs (570 upregulated genes and 366 downregulated genes) between the AKI and healthy control groups (Supplementary Table 3). Furthermore, 60 DEGs (25 upregulated genes and 35 downregulated genes) were identified in TECs between the DKD and healthy control groups (Figure 5A, Supplementary Table 4). Recent studies have reported that programmed cell death mechanisms, including ferroptosis, senescence, autophagy, apoptosis, cuproptosis, and pyroptosis, are involved in the pathogenesis of both AKI and DKD [44,45]. Next, an extensive database search focusing on genes associated with ferroptosis and pyroptosis was performed. The common DEGs were primarily associated with essential life processes, including cellular apoptosis, autophagy, and senescence.

After intersection analysis using a Venn diagram, 40 common DEGs, including 8 commonly upregulated genes (*ITGB6*, *TPM1*, *ITGB8*, *CRYAB*, *ANXA2*, *PFKFB3*, *SOX4*, and *ID1*) and 20 commonly downregulated DEGs, were identified in TECs (Figure 5H).

### 3.5. GO and KEGG pathway analysis of common DEGs in TECs

To further elucidate the potential biological functions of the common DEGs, GO analysis and KEGG pathway enrichment were performed using the R software package ‘clusterProfiler.’ The DEGs were significantly enriched in the GO terms BP, CC, and MF (Figure 5I). In the term BP, the DEGs were enriched in key pathways, such as monocyte differentiation, response to nutrient levels, cellular response to inorganic substances, and response to steroid hormones (Figure 5J). This suggests that the DEGs are involved in crucial physiological mechanisms. In the term CC, the DEGs were enriched in structures, including the apical plasma membrane, apical part of the cell, basement membrane, and cell basal part, indicating their localization and functional relevance within specific cellular compartments (Supplementary Table 5).

In the term MF, the DEGs were enriched in secondary active transmembrane transporter activity, anion transmembrane transporter activity, active transmembrane transporter activity, and symporter activity. This suggests the role of DEGs in facilitating various molecular transport processes across cellular membranes.

Additionally, KEGG pathway analysis revealed that the DEGs were significantly enriched in pathways, such as mineral absorption, focal adhesion, hypertrophic cardiomyopathy, and dilated cardiomyopathy (Figure 5K). These findings reveal the potential implications of DEGs in TECs, suggesting their involvement in crucial physiological and pathological processes. In summary, the results of the comprehensive analysis provide valuable insights into the biological functions and pathways associated with the identified DEGs, especially their role in TECs and their potential implications in various physiological and pathological contexts (Supplementary Table 6).

### 3.6. Construction and identification of hub genes in the PPI network of common DEGs

**Figure 6.**
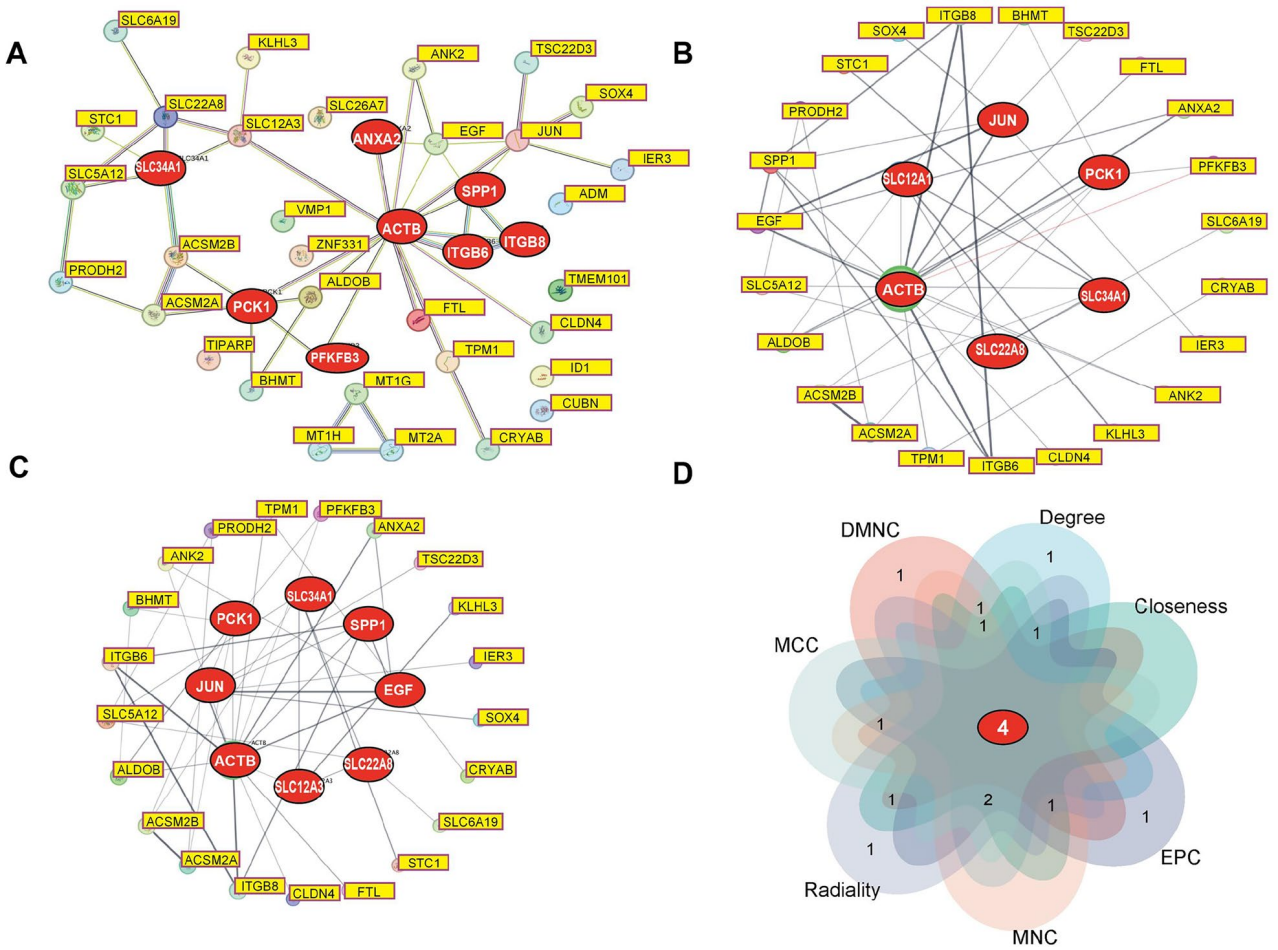
PPI Network and identification of hub genes. (A) PPI network of common DEGs between the AKI and DKD groups. The PPI network has 40 nodes and 47 edges. The PPI network was generated using the Search tool for the Retrieval of Interacting genes (STRING) database and visualized using Cytoscape. (B) The betweenness procedure of the CytoHubba plugin was implemented to obtain hub genes. The circle nodes indicate the highlighted top 7 hub genes and their interactions with other molecules. (C) The degree procedure of the CytoHubba plugin was implemented to obtain hub genes. The circle nodes indicate the highlighted top 8 hub genes and their interactions with other molecules. (D) The Venn diagram revealed 6 overlapping hub genes among the results of the seven algorithms. The circle nodes represent DEGs, while the edges represent the interactions between nodes.

To examine the direct interactions between proteins encoded by the common DEGs and identify hub genes, a PPI network of these DEGs was constructed using the STRING database and visualized using Cytoscape software. The PPI network revealed an intricate interplay among genes exhibiting common expression alterations in TECs and comprised 40 nodes and 47 edges (*p* = 5.1e-11) (Figure 6A). To identify genes involved in the pathogenesis of both AKI and DKD, betweenness analysis was performed using the Cytoscape plugin cytoNCA (Figure 6B). Node sizes were determined based on betweenness centrality. The analysis revealed close interactions among DEGs within TECs and identified the following five hub genes: *ACTB*, *SLC12A3*, *JUN*, *PCK1*, and *SLC34A1*. Additionally, degree analysis was performed using cytoNCA with node sizes representing node degree, reflecting the number of connections each node maintains within the network. This analysis identified the following eight hub genes: *ACTB*, *JUN*, *PCK1*, *SLC34A1*, *SPP1*, *EGF*, *SLC22A8*, and *SLC12A3* (Figure 6C). Furthermore, to account for the heterogeneous nature of biological networks, the CytoHubba plugin was used to concurrently utilize multiple topological analysis algorithms for hub gene identification. Seven algorithms (MCC, MNC, degree, DMNC, closeness, radiality, and EPC) were used to predict the top 10 important hub genes within the PPI network (Table 1). The intersection of these 10 genes across the 7 algorithms revealed the following four hub genes: *ITGB6*, *JUN*, *SPP1*, and *EGF* (Figure 6D).

**Table 1.** The top 10 hub genes rank in CytoHubba.

MCC	MNC	Degree	DMNC	Closeness	Radiality	EPC
*ACTB*	*PCK1*	*ACTB*	*ITGB6*	*PCK1*	*PCK1*	*PCK1*
*ITGB6*	*ACTB*	*ITGB6*	*SLC22A8*	*ACTB*	*ACTB*	*ACTB*
*SLC22A8*	*ITGB6*	*SLC22A8*	*ANXA2*	*ITGB6*	*ITGB6*	*ITGB6*
*SLC12A3*	*SLC22A8*	*SLC12A3*	*PFKFB3*	*SLC12A3*	*SLC12A3*	*SLC12A3*
*JUN*	*ALDOB*	*SLC5A12*	*ALDOB*	*PFKFB3*	*PFKFB3*	*ANK2*
*ITGB8*	*JUN*	*JUN*	*ITGB8*	*ALDOB*	*TPM1*	*ALDOB*
*SLC34A1*	*ITGB8*	*SLC34A1*	*JUN*	*JUN*	*ALDOB*	*JUN*
*SPP1*	*SLC34A1*	*SPP1*	*SLC34A1*	*SLC34A1*	*JUN*	*ITGB8*
*EGF*	*SPP1*	*EGF*	*SPP1*	*SPP1*	*SPP1*	*SPP1*
*PCK1*	*EGF*	*PCK1*	*EGF*	*EGF*	*EGF*	*EGF*

MCC: Mathews Correlation Coefficient; MNC: maximum neighborhood component; DMNC: density of MNC; EPC: edge percolated component.

These hub genes play crucial roles within the PPI network, reflecting their potential impact on relevant biological processes and disease mechanisms. In addition to providing a comprehensive understanding of the direct interactions between the proteins encoded by the common DEGs, this integrated approach identified key hub genes potentially involved in the pathogenesis of both AKI and DKD and offered valuable insights into the molecular mechanisms of these renal disorders.

### 3.7. Analysis of gene-associated regulatory networks

**Figure 7.**
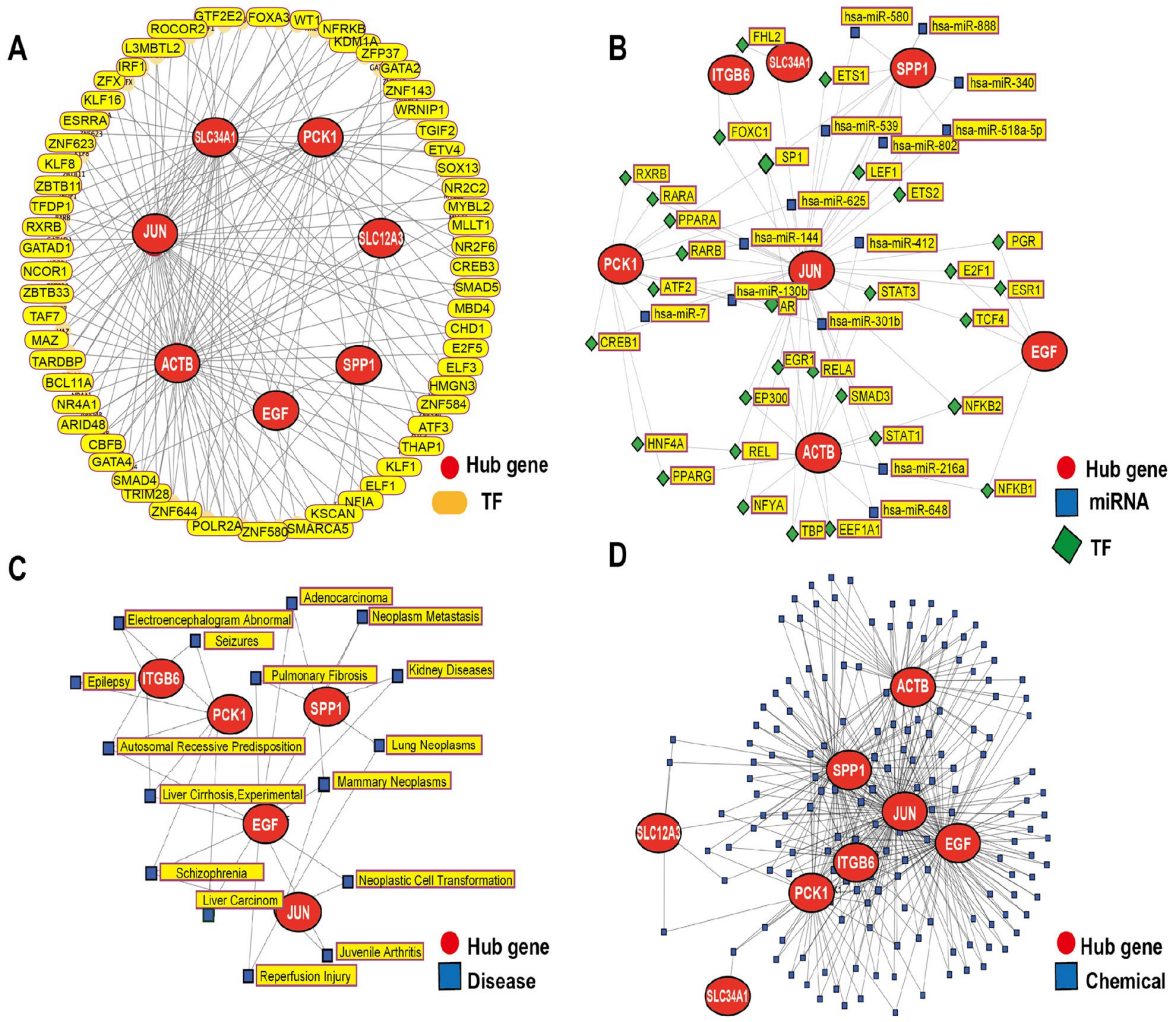
Analysis of gene-associated regulatory networks. (A) Transcription factor (TF)–DEG interaction network analysis. (B) The TF-miRNA-mRNA regulatory network. Red nodes, blue squares, and green squares represent hub genes, miRNAs, and TFs, respectively. (C) The gene-disease association network represents diseases associated with common DEGs. (D) The protein-chemical association network represents chemicals associated with common DEGs.

The PPI networks of the AKI and DKD groups were used to identify the following eight hub genes: *ITGB6*, actin beta (*ACTB*), Jun proto-oncogene, AP-1 transcription factor subunit (*JUN*), phosphoenolpyruvate carboxykinase 1 (*PCK1*), solute carrier family 34 member 1 (*SLC34A1*), secreted phosphoprotein 1 (*SPP1*), epidermal growth factor (*EGF*), and solute carrier family 12 member 3 (*SLC12A3*). To examine the functional roles of these hub genes in the TF networks, the NetworkAnalyst online tool was used to predict TFs regulating the expression of the eight hub genes and construct a comprehensive hub gene-TF network. Among these hub genes, seven were regulated by specific TFs. A network encompassing 77 nodes, and 164 edges was constructed in which the node size indicated the node degree (Figure 7A). Furthermore, the interactions within the hub gene-TF-miRNA triad were examined. In this visualization, hub genes, TFs, and miRNAs are depicted as red circles, green squares, and blue squares, respectively. Genes demonstrating robust interactions with other network components appeared larger relative to other nodes. Thus, a network comprising 52 nodes, and 96 edges was obtained. Prominent red nodes, such as *JUN* and *ACTB* exhibited enhanced significance within the network (Figure 7B).

Based on the potential pivotal role of hub genes, their interactions with diseases and small molecule compounds were examined to offer insights into potential therapeutic avenues. The NetworkAnalyst online tool was used to predict diseases associated with the eight hub genes. Next, an mRNA-disease-related network was constructed, identifying five hub genes and 16 diseases interconnected within the network (Figure 7C). Additionally, compounds linked with hub genes were examined, and a protein-chemical interaction network was constructed. This network delineated associations between eight proteins and 161 small molecule compounds and comprised 169 nodes and 431 edges (Supplementary Table 7). The red nodes, including JUN, SPP1, and ITGB6, exhibited enhanced significance within the network (Figure 7D).

### 3.8. Experimental validation of hub genes in the mouse models and HK-2 cells

**Figure 8.**
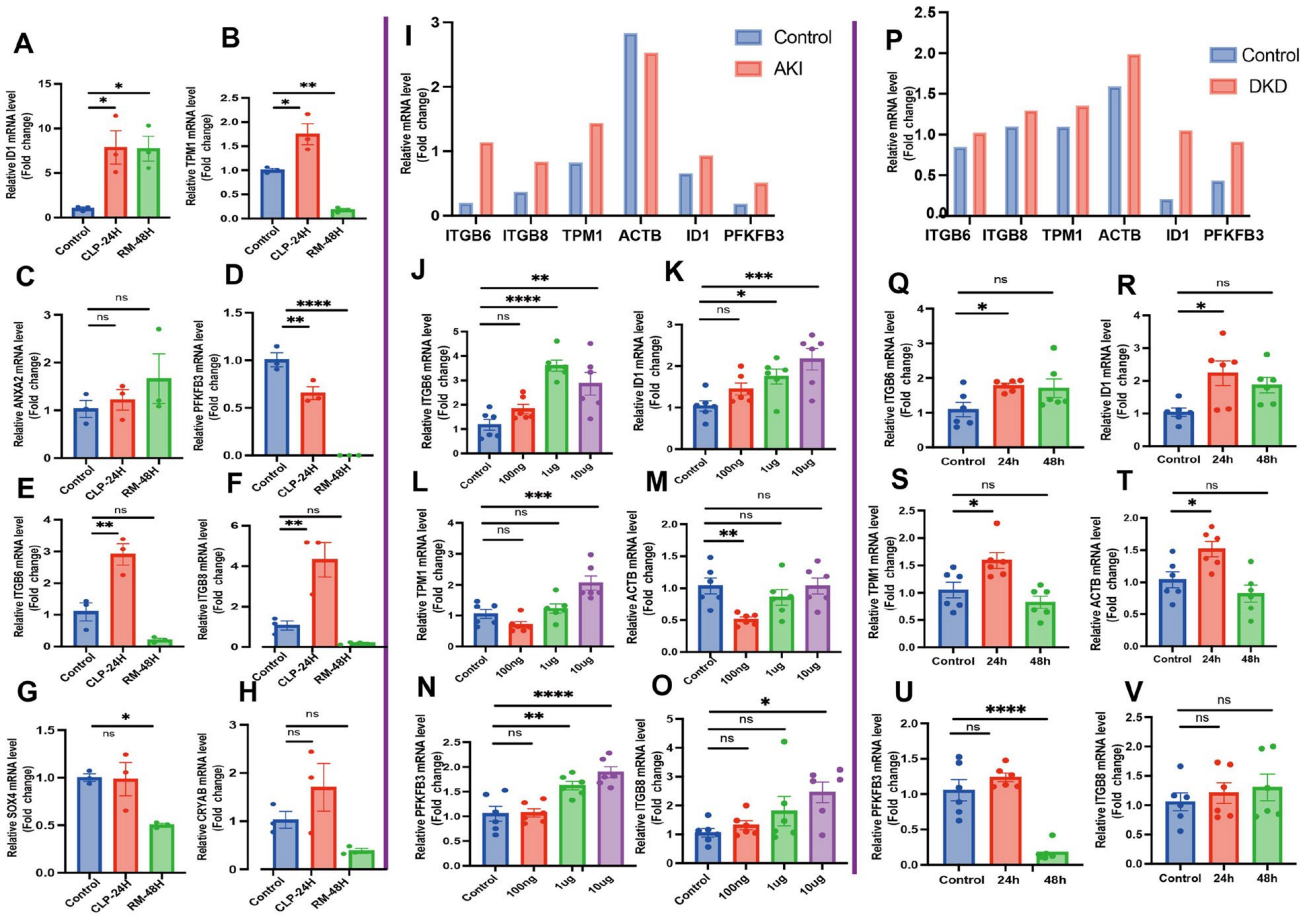
Experimental validation of hub genes in mice and HK-2 cells. (A–H) In the animal model, the relative mRNA expression levels of 8 genes in CLP-induced AKI and RM-AKI models were assessed using qRT-PCR analysis. (I, P) The relative mRNA expression of 6 genes in the DKD and AKI groups was determined using uniform manifold approximation and projection (UMAP). (J–O, Q–V) the relative mRNA expression levels of 6 genes in the DKD and AKI cell models were determined using qRT-PCR analysis. *p < 0.05, **p < 0.01, ***p < 0.001.

The eight consistently upregulated DEGs (*ITGB6*, *TPM1*, *ITGB8*, *CRYAB*, *ANXA2*, *PFKFB3*, *SOX4*, and *ID1*) were validated in mouse models. An experimental model for sepsis-induced AKI was established using CLP. Meanwhile, an RM-AKI model was established using glycerol injection. The expression levels of *Tpm1*, *Itgb6*, *Itgb8*, and *Id1* were significantly upregulated in the experimental models (Figure 8A–H). Additionally, the expression levels of these genes in the sepsis-induced AKI model were higher than those in the RM-AKI model.

An *in vitro* cell model for sepsis-induced AKI was established using LPS. This study focused on five key genes (*TPM1*, *ITGB6*, *ITGB8*, *ID1*, *ACTB*, and *PFKFB3*). In the GSE183276 and GSE131822 datasets, the TEC expression levels of *TPM1*, *ITGB6*, *ITGB8*, *ID1*, and *PFKFB3* in the AKI and DKD groups were upregulated when compared with those in the control group (Figure 8I–P). To validate the expression of these genes in disease contexts, *in vitro* experiments were performed using HK-2 cells. The expression levels of *ITGB6*, *ID1*, *TPM1*, *ITGB8*, and *PFKFB3* in the LPS-treated cells were significantly upregulated when compared with those in the control cells (Figure 8I–O). Meanwhile, the expression levels of *ITGB6*, *ID1*, *TPM1*, and *ACTB* were significantly upregulated in the glucose-treated cells (DKD model) (Figure 8P–V). Thus, the expression levels of *ID1*, *TPM1*, *ITGB8*, and *PFKFB3* were upregulated in both AKI and DKD *in vitro* models, suggesting that these genes mediate a molecular mechanism shared between AKI and DKD (Supplementary Table 8).

## 4. Discussion

Patients with DKD are susceptible to developing AKI, which can manifest acutely and impede short-term recovery or exacerbate the long-term trajectory of renal dysfunction [50]. T2DM with or without CKD is reported to be an independent predisposing factor for AKI [51,52]. In a large prospective cohort study spanning 12 years and involving Chinese patients with T2DM, the occurrence and severity of initial AKI episodes were systematically demonstrated to be significantly correlated with long-term renal complications and mortality rates [53]. Previous studies have investigated the correlation between CKD and AKI, encompassing diverse study cohorts, such as the elderly, hospitalized, and postoperative patients with a spectrum of CKD etiologies [54–57]. However, limited studies have examined the correlation between AKI and DKD or diabetes [15,16, 58]. Although AKI is a risk factor for the progression of DKD, the precise molecular mechanisms underlying the correlation between AKI and DKD have not been elucidated.

TECs, which are the predominant components of the renal cortex, are the principal target cells in the onset and progression of AKI. This can be attributed to the diminished tissue perfusion and oxygenation along with the accumulation and concentration of various noxious substances within these cells [59]. Additionally, tubular injury manifests early in DKD and regulates renal disease progression [49, 60]. Najafi et al. analyzed renal biopsy samples of patients with type 1 diabetes and reported that even with physiological or mildly impaired glomerular filtration rate, 17% and 51% of glomeruli exhibited tabularization and atrophic tubules, respectively. These clinical and pathological findings indicate the critical role of tubular injury in DKD development, potentially preceding and interacting with functional changes in glomeruli.

This study utilized bioinformatics methodologies to explore common DEGs between the AKI and DKD groups and identify potential signaling pathways and key genes involved in the interplay between these conditions. In particular, this study identified 8 consistently upregulated genes and 20 downregulated genes. GO enrichment analysis revealed that the DEGs were enriched in processes, such as mononuclear cell differentiation, response to nutrient levels, reaction to inorganic substances, and sensitivity to steroid hormones. Furthermore, KEGG analysis revealed that the DEGs were primarily enriched in pathways related to mineral absorption, focal adhesion, hypertrophic cardiomyopathy, and dilated cardiomyopathy. Furthermore, analysis of PPI networks and various gene-related networks revealed the following eight central hub genes: *ITGB6*, *ACTB*, *JUN*, *SPP1*, *EGF*, *SLC34A1*, *SLC12A1*, and *PCK1*. These genes are reported to play pivotal roles in the pathogenesis and progression of AKI and DKD. Experimental validation experiments demonstrated that the expression levels of *ITGB6, ID1*, *TPM1*, *ITGB8*, and *PFKFB3* were upregulated in the AKI and DKD *in vitro* models. We hypothesized that these four DEGs mediate a molecular mechanism shared between DKD and AKI pathogenesis and that their expression is upregulated when patients with DKD develop AKI. The expression of *ITGB6*, a hub and commonly upregulated gene, was upregulated in the *in vitro* models. Thus, *ITGB6* may serve as a biomarker for early AKI diagnosis in patients with DKD and as a potential target for early therapeutic intervention.

ITGB6 belongs to the integrin family of proteins, which are cell surface transmembrane glycoprotein receptors composed of non-covalently linked α and β subunits. These integrins play critical roles in mediating cell adhesion and migration by facilitating connections between cells and the extracellular environment [61–63]. In humans, *ITGB6* is located on chromosome 2q24-q31 [64]. ITGB6 forms a heterodimer with integrin αv, encoded by *ITGAV* located on chromosome 2q31-q32, resulting in the formation of integrin αvβ6 [65,66]. Integrin αvβ6 expression is primarily restricted to epithelial cells and is typically downregulated in most healthy tissues but frequently upregulated in various conditions, such as development, wound healing, cancer, and fibrosis [67–70].

ITGB6 regulates epithelial remodeling during development, tissue repair, and tumor formation. The integrin family members, including ITGB6, mediate the activation of transforming growth factor-beta (TGF-β), a major fibrogenic cytokine. Therefore, the interaction between epithelial cells expressing ITGB6 and stromal cells, which regulate fibrosis processes in various organs, is crucial for initiating fibrosis [71,72]. Activated TGF-β1 is a key factor mediating the pathogenesis of pulmonary fibrosis. In fibrotic lung tissue, the balance between TGF-β1 activation and ITGB6 expression is dysregulated. Consequently, the inhibition of ITGB6-mediated TGF-β1 activation is a promising therapeutic approach for fibrosis [73]. Recent studies have indicated that integrin αvβ6 promotes fibrosis in various organs, such as the lungs, liver, intestine, and kidney [72, 74–76]. In DKD, the expression of integrin β6 is upregulated in the proximal tubules of db/db mice and fibronectin-induced renal proximal tubule cells. Integrin β6 upregulation is correlated with a significant increase in epithelial-mesenchymal transition (EMT) between the epithelium and stroma. Furthermore, the urinary levels of integrin β6 are markedly upregulated in patients with DKD, indicating its pivotal role in mediating EMT in proximal TECs and offering novel avenues for the diagnosis and management of DKD. Additionally, ITGB8 exerts regulatory effects on tumor cell proliferation and invasion and tumor growth by modulating the adhesion plaque signaling pathways [77–79]. These adhesion plaques, which are predominantly regulated by integrins, establish vital connections between the extracellular matrix and the actin cytoskeleton, regulating various cellular functions [80–82]. These findings suggest that ITGB6 is a potential diagnostic marker and a potential therapeutic target for fibrotic processes associated with DKD and AKI.

PFKFB3 is a crucial regulator of glycolysis, modulating the cellular levels of fructose-2,6-bisphosphate [83]. Recent studies have reported the widespread tissue expression of PFKFB3 and its involvement in various physiological processes, such as tumorigenesis, metastasis, diabetes-related organ damage, and angiogenesis. Under physiological conditions, the expression of PFKFB3 is downregulated in diverse cell types. PFKFB3 actively stimulates glycolysis through the allosteric activation of PFK-1. The role of PFKFB3 is indispensable for fundamental cellular processes, including growth, differentiation, and functionality [84–86]. During sepsis, PFKFB3 expression is rapidly upregulated, leading to its phosphorylation and subsequent activation (at approximately 6 h post-LPS stimulation). This enhanced PFKFB3 activity increases the glycolytic flux, contributing to inflammatory damage [87]. LPS induces aerobic glycolysis in lung fibroblasts *via* the PI3K-Akt-mTOR/PFKFB3 pathway. This process further stimulates collagen synthesis, exacerbates endothelial cell inflammation implicated in pulmonary fibrosis, and enhances lung fibroblast proliferation [88,89]. The inhibition of PFKFB3 is a promising strategy for mitigating inflammatory damage, improving sepsis prognosis, and suppressing fibrosis progression. Moreover, renal inflammation is a pivotal pathophysiological hallmark of DKD. PFKFB3 inhibition ameliorates renal inflammation in the DKD mouse model [90]. Thus, PFKFB3 is a potential biomarker and a therapeutic target for inflammation-induced damage and fibrosis in AKI and DKD.

ID1, a helix-loop-helix (HLH) protein, forms heterodimers with the members of the basic HLH family of TFs. Although ID1 lacks DNA binding activity, it exerts inhibitory effects on the DNA binding and transcriptional activities of basic HLH proteins upon direction interaction [91]. Previous studies have proposed the potential role of ID1 in cellular processes, such as growth, aging, and differentiation [92,93]. Recent studies have reported that ID1 is primarily upregulated in TECs during renal ischemia/reperfusion (I/R) injury in rats, as well as during hypoxia/reoxygenation (H/R) in an *in vitro* H/R model [94]. Moreover, ID1 is reported to regulate capillary growth and remodeling following I/R injury. The overexpression of ID1 reduces capillary rarefaction, enhances peritubular fibroblast proliferation, and exacerbates fibrosis [95], suggesting a pivotal role of ID1 in AKI pathogenesis. Although studies on the role of ID1 in DKD are limited, ID1 is reported to be a promising therapeutic target for patients with both AKI and DKD.

Tropomyosin 1 (TPM1) is a member of the tropomyosin family, which is a group of ubiquitous proteins with actin-binding activities. This family of proteins regulates the contraction mechanisms in both striated and smooth muscle tissues and modulates the cytoskeletal architecture in non-muscle cells. In addition to contributing to cytoskeletal stability, TPM1 mediates diverse physiological phenomena, including cytokinesis, cellular motility, apoptosis, and activation of signal transduction pathways [96]. Previous studies have reported the significance of TPM-encoding gene mutations in muscle cells, correlating them with a spectrum of muscular disorders, such as familial hypertrophic cardiomyopathy, severe myasthenia gravis, and arterial occlusive diseases [97–99]. Tumorigenesis and tumor progression are associated with the dysregulation of TPM expression [100]. TPM1 is characterized as a tumor suppressor and can induce apoptosis in cancer cells, suppressing cancer progression [101]. Despite these advancements, the role of TPM1 in the pathogenesis of AKI and DKD is unclear, warranting further investigation.

However, this study has several limitations. First, the number of patients in the DKD dataset was relatively small. Thus, the sample size must be increased to effectively mitigate individual variabilities. Second, biopsy specimens from a part of the kidney may not completely represent the pathological changes occurring in the entire organ owing to the diverse nature of injury types in AKI and the heterogeneous nature of renal damage. Third, although the mRNA expression levels were validated in patients with both AKI and DKD, further animal studies and validation at the protein level are warranted to corroborate the findings of this study. These limitations will be addressed in future experimental studies.

## 5. Conclusions

This study signifies a pioneering application of the combination of scRNA-seq data and bioinformatics analyses to explore the role of TECs in the AKI and DKD models. Validation studies with the DKD and AKI models revealed that *ITGB6, ID1*, *TPM1*, *ITGB8*, and *PFKFB3* mediate a molecular mechanism shared between DKD and AKI. In particular, the expression of *ITGB6*, a hub and commonly upregulated gene, was upregulated in the *in vitro* models. *ITGB6* may serve as a biomarker for early AKI diagnosis in patients with DKD and as a potential target for early therapeutic intervention. The findings of this study provide novel insights for the diagnosis and treatment of AKI and DKD.

Based on the data analysis in this study, we suggest the initiation of comprehensive clinical investigations involving patients and fundamental research using animal models. These endeavors may elucidate the molecular mechanisms shared between DKD and AKI and consequently aid in the development of novel therapeutic interventions for these conditions.

## Supplementary Material

Supplementary8.xlsx

Supplementary2.xlsx

Supplementary3.xlsx

supplementary figure1.png

Supplementary5.xlsx

Supplementary4.xlsx

Supplementary6.xlsx

Supplementary7.xlsx

Supplementary1.xlsx

## Data Availability

All materials are owned by the authors and/or no permissions are required. If you need more details, please contact Heng Jin (E-mail: hengjin@tmu.edu.cn).
