# Supplementary figures and images for: Integrin subunit beta 6 is a potential diagnostic marker for acute kidney injury in patients with diabetic kidney disease: a single cell sequencing data analysis
Source: Ren Fail. 2024 Oct 2;46(2):2409348. doi: 10.1080/0886022X.2024.2409348 (PMC11448326; doi:10.1080/0886022X.2024.2409348)

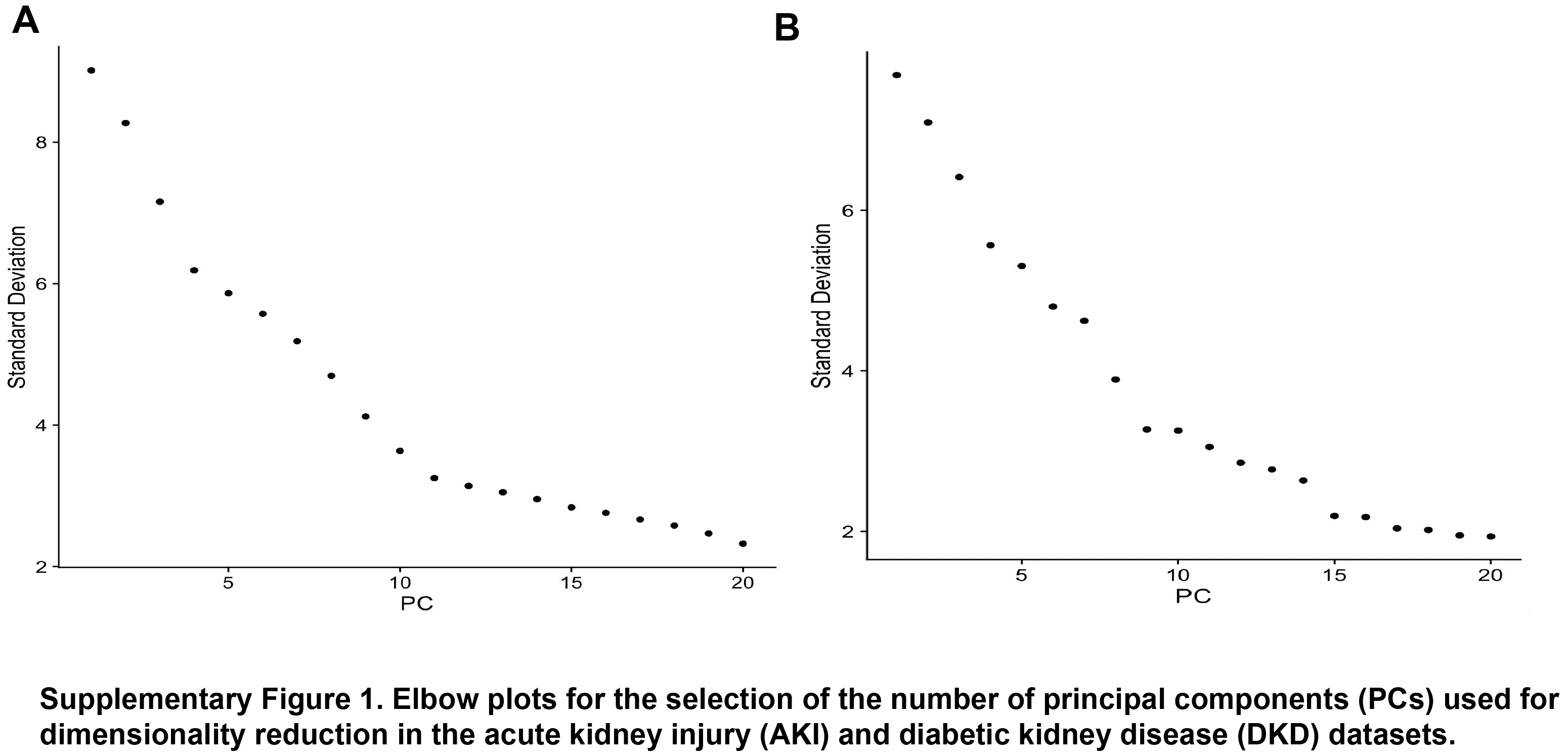

Supplement: supplementary figure1.png [file IRNF_A_2409348_SM4855.png]
